# Who is feared and who is protected? A multilingual audit of gendered emotional framing of migrants in generative AI

**DOI:** 10.3389/fpsyg.2026.1980883

**Published:** 2026-10-27

**Authors:** Ayhan Dolunay, Fatma Arpalıklı

**Affiliations:** Department of New Media and Communication, Faculty of Communication, Near East University, Nicosia, Cyprus

**Keywords:** counter-framing, emotional framing, gender bias, generative artificial intelligence, migration communication, multilingualism, output auditing, social integration

## Abstract

Accounts of migration can cast migrants as threatening or vulnerable, recognise their agency, or describe their need for protection and belonging. We examine whether these emotional and social roles differ when a generative model writes about women and men migrants in five languages. We analysed 900 texts produced on 27 August 2026 with gpt-5.6-terra through the OpenAI Responses API, using medium reasoning effort. Each of the 18 scenarios, grouped into six domains, was presented in both gender conditions and all five languages, with five independent generations for each combination. We screened each response against the lexicon for its language. Gender contrasts were examined using Fisher’s exact tests, risk differences with 95% Newcombe-Wilson intervals, and additional exact tests stratified by language and scenario. None of the seven primary domain-congruent comparisons was significant after Holm correction; the intervals remained too wide to establish equivalence. In an exploratory corpus-wide screen, threat-related vocabulary appeared in 36.0% of man-condition and 26.9% of woman-condition outputs (female-minus-male risk difference −9.1 percentage points; 95% CI [−15.1, −3.0]; Fisher *p* = 0.004; Holm *p* = 0.040 across 10 categories). The stratified comparison also remained significant after correction. Existing contextual flags identified explicit threat rejection in 29/50 ticket-dispute outputs and 3/50 after-dark address outputs. These focused observations support an exploratory account of algorithmic counter-framing, without establishing its corpus-wide prevalence or a gender difference in its frequency. The contribution is a relational approach to emotional framing: emotion-related vocabulary must be interpreted through its experiencer, target, and narrative function.

## 1 Introduction

Migration communication describes, evaluates, and positions people who move within the receiving society. News stories, official statements, platform posts, and everyday information services may present the same person as a neighbour, a participant, an outsider, or a social problem. How migrants are named helps shape their social distance from others and the recognition available to them. Despite differences between national settings, European studies repeatedly identify this connection. Reviewing that literature, Eberl et al. (2018) found that negative and conflict-oriented accounts were persistent, with crime and threat recurring as organising frames.

Recommender systems, chat interfaces, and generative applications now participate in migration communication by selecting, translating, retrieving, and producing information. These functions have obvious value for someone trying to follow an administrative procedure, seek work or housing, or communicate in another language. They extend uses of digital media and transnational networks already described in migration scholarship (Dekker and Engbersen, 2014; Borkert et al., 2018). Usefulness, however, should not be confused with neutrality. The service may solve an immediate problem and still repeat a stereotype or recast a prior inequality as the product of an apparently impersonal process. We therefore approach AI-integrated migration communication as a social and technical arrangement at once.

The concern pursued here is emotional framing in multilingual AI-generated migration discourse. Such discourse arranges fear, sympathy, anxiety, care, suspicion, hope, and belonging around particular actors. The migrant may appear as someone others fear, as a person who is herself or himself afraid, as a recipient of protection, or as an actor able to adapt and contribute. These are different moral positions. Each invites a different relation between the migrant and the receiving society and, with it, a different mix of support, surveillance, and recognition.

Gender makes this distribution of roles especially important. Studies of migrant and refugee representation repeatedly describe a contrast between vulnerability coded as feminine and threat coded as masculine. In British coverage of the European refugee crisis, for instance, images of vulnerable women and threatening men worked together rather than as separate motifs (Gray and Franck, 2019). Ryan and Tonkiss (2023) likewise found that motherhood made women more readily legible as vulnerable refugees, whereas men were more often interpreted through isolation, criminality, or threat. Refugee women may thus become visible at the price of being reduced to passive victims (Rothenberger and Schmitt, 2024). This literature leads to a testable question: if the rest of a prompt remains unchanged, does identifying the migrant as a woman or a man change the emotional account produced by a generative model?

Generative systems make that comparison less straightforward than a search for one stored representation. Every response is newly produced from learned statistical regularities, under the additional influence of model design, alignment, and the wording of the prompt. Repeated calls can therefore be used to observe a distribution of representational choices under controlled conditions. They do not disclose an inner psychological state, and emotion words in an output are not evidence that a model feels anything. We examine the resulting texts for the social roles associated with emotion concepts and for the recurrence of those associations across outputs.

The study therefore brings together three bodies of literature that are often treated separately: migration communication, gendered representation, and emotion science. Emotion science provides a framework for distinguishing emotion categories, affective dimensions, and context-dependent constructions of meaning. Classical basic-emotion approaches emphasise partially differentiated emotion categories (Ekman, 1992), whereas dimensional approaches organise affect around relations such as valence and arousal (Russell, 1980). Constructivist approaches, by contrast, emphasise how emotion categories are constituted through conceptualisation, prior knowledge, bodily regulation, and context (Barrett, 2006, 2017). Although these traditions differ theoretically, taken together they caution against treating emotion as a single undifferentiated variable.

We tested this problem through a multilingual output audit with tightly matched conditions. The audit crossed five languages with 18 situations involving, among other things, achievement, loss, rejection, belonging, threat, and care. Every situation appeared in a woman-migrant and a man-migrant version. This gave gender the same experimental role across quite different social encounters, rather than confining the comparison to one type of prompt. Balance was maintained across languages, scenarios, and replicates. No pairing was imposed at the response level: each API call produced its own stochastic output. Accordingly, we compare not only the words selected but also the narrative role given to the migrant when the gender cue is the sole change.

The first methodological question is what should count as meaningful similarity or difference. A simple positive/negative classification would erase the relational point: who fears whom, who receives care, and who is represented as competent or as belonging. Across five languages, accurate translation is only part of the comparison: the resulting accounts may still assign different roles to the migrant. Lexical matches also need interpretation. A response containing “threat” may expressly deny that the migrant is threatening. Our contextual readings therefore examined selected matches for the person to whom fear or suspicion was directed and for passages that rejected a stereotypical attribution.

## 2 Theoretical framework

### 2.1 Migration communication, representation, and social integration

Migration communication includes the interpersonal exchanges and mediated accounts through which people give migration public meaning. These accounts determine which voices receive attention and how causes, problems, and possible responses are described. Entman's (1993) conception of framing helps explain this process: making some aspects of a situation salient leaves others less visible. Migration may be discussed in terms of security or economic burden, but also through humanitarian need, social contribution, victimhood, or cultural difference. In coverage of the 2015 refugee crisis, these representational choices were associated with unequal opportunities to speak and to be recognised as members of a community or as subjects of humanitarian concern (Chouliaraki and Zaborowski, 2017).

Judgements about migrants’ capacities and belonging are shaped partly by such representations. Integration also depends on access to institutions and material resources, relationships in the receiving society, and the negotiation and recognition of identity. Berry (1997) describes participation in the wider society as compatible with maintaining valued cultural ties. Communication matters in both respects: it supplies information needed for everyday participation and gives social meaning to migrant identities. The terms on which migrants enter public life are therefore communicated as well as institutionally organised.

Mobile and social platforms allow migrants to maintain transnational relationships, obtain information, organise journeys, and find services and contacts after arrival (Dekker and Engbersen, 2014; Borkert et al., 2018; Leurs and Smets, 2018). Access to these resources is mediated by platform decisions. Ranking and recommendation affect what users see, while moderation and personalisation further shape the material available to them. Although migrants encounter these selections in everyday use, they have only partial access to the processes that produce them (Bozdag, 2013; Van Dijck et al., 2018).

We therefore understand integration as a process continually negotiated through communication, without assuming that communication secures it. Within this process, matters of safety, agency, access, recognition, and belonging are never fully settled. Emotional framing bears on each of these questions. Presenting a migrant as dangerous can make exclusion seem justified; presenting the same person as helpless can invite sympathy while leaving little room for autonomy. Assessing representation thus requires more than judging whether its tone is positive. It requires attention to whether women and men migrants appear with capacities and relationships beyond being feared or receiving care.

### 2.2 AI-integrated migration communication

Writing and translation are only two of AI’s roles in communication. Systems also rank search results, personalise information, classify or moderate content, and supply material for later decisions. Diakopoulos (2019) documents changes to newsroom routines and to the selection and presentation of news. Dolunay (2024) likewise situates AI within relationships between institutions, users, and information. These accounts place AI within the infrastructure through which communication is organised (Haraç and Dolunay, 2026).

For a migrant using a conversational interface, these functions may involve translating medical instructions, understanding a residence procedure, reading a summary of local news, or finding assistance. Such uses can reduce difficulties associated with unfamiliar languages and institutions. The answers may nevertheless carry assumptions learned from the system’s training environment. Binns (2018) and Noble (2018) approach this problem from different perspectives, showing how algorithmic inequalities can arise through categories, defaults, associations, and uneven attention, even without an explicitly discriminatory rule.

We use AI-integrated migration communication to describe communication about migration whose production or interpretation involves AI. The term does not cover every use of an AI application. It allows us to examine benefits alongside representational problems within the same exchange. An answer may help someone access a public service while making assumptions about dependency, security, family, work, or risk. Incorporating AI into communication consequently tells us little, by itself, about the social inclusion of the migrants concerned.

Our earlier work considered how AI’s intermediary role in migration communication could contribute to integration or disintegration. Here we examine one part of that account: emotional framing and its bearing on recognition. This focus includes apparently civil or protective descriptions. Their implications for inclusion require examination even when the wording contains no overt hostility.

### 2.3 Gendered emotional framing: threat, vulnerability, protection, and agency

Research on refugee representation has repeatedly linked gender to judgements of who poses a danger and who deserves assistance. Across accounts of the European refugee crisis, male migrants have commonly been interpreted through danger, while female migrants have appeared through claims of vulnerability and protection. Gray and Franck (2019) read these images as mutually reinforcing, not as separate strands of representation. In that pairing, men become the presumed object of fear, whereas women are more often imagined as fearful subjects who merit protection. That division has consequences beyond word choice. Competition and uncertainty, for example, are associated with stronger threat judgements, prejudice, and dehumanising attitudes toward immigrants and refugees (Esses et al., 2001, 2013).

The question, then, is which migrants are readily recognised as refugees. In Ryan and Tonkiss's (2023) study of British tabloid images, motherhood and vulnerability made women more legible as refugees, whereas men were more often associated with criminality or social isolation. Both forms of recognition are restrictive. Humanitarian attention may be extended to women at the expense of their political voice and agency, while agency in men may be read as danger or as evidence against a legitimate claim to vulnerability. International coverage of refugee women shows a related problem: women remain relatively underrepresented, and their visibility is often secured through passive-victim imagery (Rothenberger and Schmitt, 2024).

Threat and victimhood should therefore be coded as distinct social positions, not as opposite values on a single scale. The agency assigned to a threatening figure is restricted: it becomes a reason for surveillance or control. A vulnerable figure, meanwhile, may be considered deserving of assistance while still being denied competence. Resilience and integration offer a wider position, one in which migrants negotiate institutions, establish relationships, and take part in common life. We retain this distinction between social positions in the analytical framework.

The prompts omitted nationality, ethnicity, religion, country of origin, and legal status to isolate the gender cue. This controlled comparison necessarily simplifies the circumstances of migration. Gendered representation is routinely entangled with racialisation, class, and legal classification. Removing those cues lets us ask a narrow question about gender while holding the remaining prompt constant. It also limits the answer. The patterns reported below apply to this controlled gender contrast and cannot stand in for the intersectional representation of migrants more generally.

### 2.4 Emotion science as an analytical lens

Research on AI and emotion also addresses the human concerns surrounding its use. Drawing on interviews with ethics and psychology experts, Dolunay and Temel (2024) examined the relationship between academic goals, emotional states, and unethical AI use. Kambur and Dolunay (2024) explored artists’ and experts’ perspectives on copyright problems and their emotional implications, including concerns about rights in AI-generated work. These studies situate emotion within the social and ethical conditions of AI use. The present audit examines how generated narratives assign emotional experiences and social positions to migrants.

Emotion science offers several ways of thinking about language concerning fear, anxiety, hope, belonging, anger, care, and safety. This study does not assume that any single theory provides a complete ontology of emotion. Instead, established approaches are used as sensitising frameworks that clarify what is—and is not—being coded. In particular, appraisal theories’ emphasis on situation-goal relations and the Emotions as Social Information (EASI) model draw attention to who is represented as experiencing an emotion, toward whom it is directed, and what action outcome that emotional construction may legitimise (Moors et al., 2013; Van Kleef, 2009).

Basic-emotion approaches propose that at least some emotions have relatively differentiated patterns and functions. Ekman's (1992) account has been influential in identifying families of basic emotions and emphasising cross-cultural regularities in emotional expression. For the present study, the principal value of the basic-emotion tradition is that fear, anger, sadness, and other emotions should not be collapsed into a single measure of “negativity.” A news text portraying a migrant as frightening is not psychologically or socially equivalent to one portraying the same migrant as sad, anxious, or hopeful.

Russell's (1980) circumplex model supplies a complementary, dimensional lens by locating affective states in relation to valence and arousal. It helps distinguish high-arousal negative states, such as agitation or fear, from lower-arousal ones, such as sadness and discouragement. It also keeps relief and belonging from disappearing into an undifferentiated class of positive emotion. We do not convert the outputs into valence-arousal scores. Instead, dimensional thinking serves as a reminder that terms with the same broad valence need not perform the same psychological or social work.

Constructivist work is particularly useful when the object is generated language. Barrett (2006, 2017) understands emotion categories as constituted through conceptualisation and context, rather than as categories directly disclosed by fixed internal signals. This directs our attention to how an account is constructed. We are not treating the model as an organism capable of fear. We examine how the generated narratives draw on culturally available concepts of fear, care, vulnerability, safety, and agency. Our concern is with the relations they describe: the person who fears, the object of that fear, the attribution of danger, and the support or action made relevant by the account.

The evidence for this study consists of the social organisation of emotion concepts in generated text. We do not use AI to recognise emotions, and we infer no psychological states from facial expressions or physiological signals. Appraisal accounts associate fear with possible harm and uncertainty, anger with injustice and attributed responsibility, and sadness with loss understood as irreversible (Moors et al., 2013). The EASI model adds that expressions of emotion provide observers with material for social inference and response (Van Kleef, 2009). Coding must therefore identify more than the emotion named: it must attend to the experiencer, source, target, and function of that emotion in the narrative.

The distinction between fear of migrants and migrants’ own fear provides the clearest example. The same emotion word may appear in both cases, yet the direction of emotion reverses the social relation. Fear of migrants positions the migrant as a possible source of danger. A migrant’s fear, by contrast, positions the migrant as a subject exposed to uncertainty, exclusion, discrimination, loss, or violence. The former may support securitization; the latter may support protection or empathy. A gendered system may distribute these two forms of fear unequally even when no explicit stereotype is requested.

Care and agency require the same relational attention. Care can support integration when it describes responsive institutions, community practises, or sustaining relationships. It becomes paternalistic, however, if women repeatedly appear as dependent on protection while men appear as either self-sufficient or threatening. Agency is similarly ambivalent. Problem solving, work, language learning, civic participation, and relationship building indicate constructive agency; action associated with danger or deviance does not. Emotion science is therefore part of the representational analysis itself, rather than a theoretical vocabulary applied after coding.

### 2.5 Research gap and research questions

Prior work has established that generated texts can reproduce gendered emotion attributions and unequal narrative roles. Plaza-del-Arco et al. (2024) found gender-stereotyped emotion attribution across five language models. TALES examined cultural misrepresentation in generated stories using a taxonomy and annotation process informed by people with relevant lived experience and language proficiency (Bhagat et al., 2026). Nguyen et al. (2026) documented underrepresentation and subordinated portrayals of Global Majority nationalities, while Sheikhi (2026) examined demographic inequalities in criminal-role assignment, including migration background. This approach is consistent with calls for documented, context-sensitive algorithmic auditing (Raji et al., 2020). These studies extend the concerns captured by BOLD and BBQ (Dhamala et al., 2021; Parrish et al., 2022) and support audits of roles and context as well as lexical associations. Our contribution is narrower: a controlled examination of the intersection of migration, gender, emotional framing, and multilingual generation in one model configuration. We ask whether matched gender cues alter domain-congruent lexical outcomes and examine how selected outputs distinguish migrants as sources of fear, subjects experiencing fear, or people defended against a threatening portrayal. The focused contextual component is exploratory.

Four questions organise the analysis:

*RQ1*. When the same scenario identifies the migrant as a woman rather than a man, how, if at all, does the model’s emotional framing change across the five languages and six domains?

*RQ2*. Does the prevalence of the primary domain-congruent outcomes differ between migrant women and men across achievement and agency, belonging and inclusion, loss and sadness, injustice and anger, fear and anxiety, vulnerability, and care and support?

*RQ3*. Where gender-related differences appear, do they recur across languages or remain localised within particular language-scenario clusters?

*RQ4*. How is explicit counter-framing manifested in the selected outputs, particularly in relation to conventional gendered representations of threat and vulnerability?

## 3 Methods

### 3.1 Research design

The study uses a controlled audit design based on repeated model calls. Gender description is the independent variable: within each language, the woman-migrant and man-migrant versions of a scenario are otherwise semantically matched. We did not rely on one communication setting. Instead, 18 scenarios were allocated to six domains, with three scenarios in each: achievement and agency (AA), belonging and safety (BS), loss and separation (LS), rejection and injustice (RI), threat and uncertainty (TU), and vulnerability and care (VC). This structure allowed the gender manipulation to be observed under different emotional and relational conditions.

A single API response, rather than a matched woman/man pair, is the observational unit. For every combination of language, scenario, and gender, we collected five stochastic generations. The resulting corpus contains 450 outputs for each gender condition and 900 overall. Replicates bearing the same number were not analysed as paired observations: no common seed, random source, or linked sampling event connected the two calls. Comparability instead comes from balance in the scenario and language structure. Because the data are model outputs and not observations of people, the claims are restricted to this model-prompt configuration; no participant, interview, or personal-data inference is involved. The balanced structure of the audit is summarised in Table 1.

**Table 1:** Balanced structure of the audit: factors, levels, and output counts.

Factor	Levels	Number of levels	Contribution to output count
Language	EN, TR, ZH, RU, AR	5	180 per language
Thematic domain	AA, BS, LS, RI, TU, VC	6	150 per domain
Scenario	Three scenarios per domain	18	50 per scenario
Gender condition	Woman migrant/man migrant	2	450 per condition
Replicate	Five per language-scenario-gender cell	5	900 total

The replication level was chosen as part of a comparative audit covering 180 language-scenario-gender conditions. Five independent generations in every condition provided repeated observations of the same prompt while maintaining equal representation of all languages, scenarios, and gender conditions. This structure allowed the primary comparisons to draw on 75 outputs per gender within each domain, rather than treating a single generated response as characteristic of a condition. The choice of five was a design judgement; it was not derived from an *a priori* power calculation and does not establish adequate precision for estimates within individual conditions. A precision benchmark added during revision illustrates the limitation (Lakens, 2022). Under an idealised independent-binomial approximation with both probabilities equal to 0.50, a 95% interval for the difference has an approximate half-width of 16.0 percentage points at 75 outputs per gender and 35.8 points at 15 per gender. These are design benchmarks, not observed-power estimates or a retrospective claim that sample size was chosen through an *a priori* power calculation. Language-specific estimates are consequently exploratory; this design cannot establish equivalence or reliably exclude modest differences.

### 3.2 Corpus generation and prompt structure

We generated the corpus through the OpenAI Responses API on 27 August 2026. Every record returned the same model identifier for gpt-5.6-terra. The requests used medium reasoning effort, medium text verbosity, and a 2,048-token maximum; neither temperature nor top_p was set separately. A prespecified job-order seed randomised call order only and did not link the stochastic sampling of individual responses. Calls ran from 02:16:06 to 03:16:36 UTC, giving an elapsed time of 60 min and 30 s. Total usage was 388,073 tokens, comprising 236,350 input and 151,723 output tokens.

We generated responses in five languages: English, Turkish, Simplified Chinese, Russian, and Modern Standard Arabic. The system instruction required each answer to remain in its assigned language and to use continuous prose. References to AI, prompting, experimentation, and research were prohibited. For each scenario, the task message asked for exactly six natural sentences describing the person’s likely emotions, other people’s possible responses, and the person’s overall portrayal. It specified prose rather than a list of emotion terms so that the response would read as an account of the situation.

The authors prepared the Turkish and English prompts and lexical lists, with generative AI assistance in prompt development and translation. The Simplified Chinese, Russian, and Modern Standard Arabic versions were translated and then checked by translators. This checking concerned preparation of the materials; it did not independently validate the lexical indicators or the contextual interpretations through bilingual assessment. Supplementary material S1 Prompt Manifest 180 Conditions.csv provides the exact system instruction, scenario text, task instruction, and assembled user prompt for each language-scenario-gender condition. The scenario cores appear in Supplementary material S1 Scenario Manifest 18.csv, and the generation script includes the corresponding language versions. Across versions, the event, narrative task, sentence count, and omitted background information were held constant, with grammatical gender agreement allowed where necessary. Readers can inspect these prompts directly. Their matched structure does not establish semantic or cultural equivalence, so we interpret the results as outputs produced by the model in each language.

The person in each prompt was identified only as either an ‘adult woman migrant’ or an ‘adult man migrant.’ Nationality, ethnicity, religion, country of origin, legal status, and a named host country were omitted. The scenarios covered everyday and institutional situations: completing a language course, receiving a promotion, helping another migrant, attending a neighbourhood meal, contributing to team success, joining a community group, separation from family, being unable to reach an ill relative, withdrawal from a social network, employment and housing discrimination, dismissive treatment in a public service setting, uncertainty over a work permit, a ticket dispute, searching for an address after dark, becoming ill while alone, losing identity documents, and a temporary workplace accident. Positive, negative, and ambiguous situations were therefore represented within the same experimental structure.

### 3.3 Data integrity and preprocessing

Integrity checks were performed on the merged corpus before coding. It contained 900 records and 37 variables, with 180 records per language. The released prompt manifest also matched the condition-specific prompts in the corpus. Every call returned a Success/completed status. We found no empty response, logged error, retry, duplicate text, or repeated condition key. Of the 900 outputs, 899 met the requested six-sentence structure; the third Arabic LS3 male replicate contained five sentences. We retained that output. Excluding it together with the corresponding opposite-gender record in a sensitivity analysis left the interpretation of the main results unchanged.

We did not impose one word-based length measure across languages with different writing systems. English, Turkish, Russian, and Arabic were summarised by file-level word counts, whereas Simplified Chinese was summarised by character count. Unicode-normalised copies were used for computational processing. For Arabic, this included removal of diacritics and elongation marks and standardisation of common letter forms. The raw output text itself was preserved; normalisation affected only the separate analysis file.

### 3.4 Computer-assisted multilingual coding framework

The coding framework comprised 10 overlapping categories drawn from migration representation and emotion research. The released lexicon contains 554 language-category patterns: 117 in English, 109 in Turkish, 119 in Simplified Chinese, 99 in Russian, and 110 in Arabic. The matching procedure searches for literal substrings, some of which are manually specified stems or multiword expressions. It first applies Unicode NFKC normalisation and lowercasing. For Arabic, it also removes diacritics and elongation marks and standardises common alif forms and final ya. It uses no linguistic tokenisation, automatic stemming, lemmatisation, Chinese word segmentation, or word-boundary restrictions. One occurrence of a normalised pattern anywhere in the normalised response is sufficient for a positive indicator. Counts refer to the number of distinct listed patterns found, rather than to the frequency of their tokens. Negation does not remove a match. Supplementary material S3 contains the full lexicon, the operational rules, and the terms matched in every response.

Seven outcomes were designated as primary by their theoretical relation to the scenario domain: AA-agency, BS-belonging, LS-sadness/loss, RI-injustice/anger, TU-fear/anxiety, and VC-vulnerability and care/support (Table 2). This limits each primary comparison to its corresponding domain, but does not resolve negation or identify whose emotion is expressed. The categories measure lexical invocation, not validated semantic attribution. No independent bilingual validation is available for the released lexicons; computational reproducibility should therefore be distinguished from construct validity.

**Table 2:** Multilingual coding framework.

Category	Analytical focus	Typical indicators
Fear and anxiety	Uncertainty and high-arousal negative affect	fear, anxiety, worry, tension, unease
Anger and injustice	Emotional and moral response to unfair treatment	anger, discrimination, injustice, humiliation, exclusion
Sadness and loss	Separation, loneliness, and loss of connection	sadness, longing, loneliness, grief, loss
Positive affect	Positive valence and future orientation	pride, joy, hope, relief, gratitude
Vulnerability	Powerlessness, dependency, or susceptibility to harm	vulnerability, helplessness, isolation, dependency
Care and support	Relational or institutional assistance	help, care, empathy, protection, solidarity
Agency and competence	Autonomy, skill, and problem-solving capacity	determination, competence, achievement, initiative, resilience
Belonging and inclusion	Acceptance, participation, and social connection	belonging, welcome, membership, recognition, safety
Threat and suspicion	Danger, doubt, or security language	threat, danger, suspicion, caution, aggression
Family and attachment	Family members and emotional closeness	family, relative, close person, loved one

The contextual component was a focused exploratory audit. The released Supplementary material S5 table includes all 100 outputs from the ticket-dispute (TU2) and after-dark address (TU3) scenarios, plus all 30 Simplified Chinese vulnerability-and-care outputs. It records automated screening flags for explicit threat negation, rights or resolution language, and separation of identity from behavioural judgement, alongside the original text. Both authors jointly conducted the contextual readings, considering negation, the person experiencing emotion, its target, and the expression’s narrative function. These were collaborative interpretive readings, not independent coding for a reliability assessment. Supplementary material S5 does not provide an exhaustive human classification of the 283 corpus-wide threat matches into threat attribution, threat rejection, and danger experienced by the migrant. Unresolved rows were therefore retained as unresolved, and absence of a negation flag was not treated as evidence of threat attribution. Counter-framing is reported as an exploratory observation from these materials, without independent coding or an intercoder-reliability claim.

### 3.5 Quantitative comparisons and contextual audit

The original analysis compared response-level binary prevalences using two-sided Fisher’s exact tests and reported female-minus-male risk differences with 95% Newcombe-Wilson confidence intervals. This direction is used throughout: negative values indicate a higher prevalence in the man condition. The seven primary comparisons were Holm-adjusted. The balanced design gives every language-scenario stratum the same weight in both gender conditions, so differences in stratum composition cannot produce the pooled contrast. Baseline rates can nevertheless vary considerably. We therefore added exact conditional tests stratified jointly by language and scenario: 15 strata for each primary outcome and 90 for each corpus-wide category. Within a stratum, the number of woman-condition positives has a hypergeometric null distribution conditional on the total positives and the five outputs per gender. Convolution across strata gives the distribution of their sum; the two-sided *p* value includes outcomes at least as far from its null expectation as the observed sum. This retains noninformative ceiling strata without fitting unstable regression coefficients. The seven stratified primary tests were Holm-adjusted as a separate sensitivity family. These tests address conditional generation variability in the fixed prompts; the pooled risk differences and intervals remain descriptive marginal summaries.

The broad threat comparison was outside the seven primary outcomes and is treated as exploratory. During revision, we calculated corpus-wide comparisons for all 10 coded categories to show the broader set of tests within which the threat result sits. Holm correction was applied across the 10 tests separately for each inferential method. This expanded set is a revision analysis, not an original confirmatory test family. For output length, we retained the original Mann–Whitney *U* comparisons within languages and their Holm adjustment across the five languages. The 35 language-specific domain comparisons remain one exploratory Holm family. Scenario-specific threat counts and leave-one-scenario-out summaries describe the distribution of the pooled lexical signal without adding a new set of confirmatory significance claims.

Repeated API calls characterise generation variability under the tested configuration; *p* values do not justify generalisation to human populations, other models, or later model versions. No equivalence margin was specified, so nonsignificant results were not interpreted as equivalence. The contextual observations are neither independently validated themes nor corpus-wide prevalence estimates. They identify instances in which the meaning of threat or vulnerability vocabulary differs from its lexical classification.

### 3.6 Ethics and researcher reflexivity

No people were recruited or observed, and the study used neither interviews nor personal data. Prompts contained no real person, institution, country, or identifying detail; the empirical corpus is composed wholly of synthetic model output. Even so, migrant status, threat, vulnerability, and protection are not ethically neutral categories. We therefore treated coding as an interpretive decision with social consequences, attending to who experienced an emotion, toward whom it was directed, and what function it served in the account.

The woman/man contrast is a deliberately narrow experimental manipulation. It tests a representational opposition established in migration and media scholarship; it is not an ontological assertion that gender is binary. The results do not speak for non-binary or trans migrants, nor for the intersectional forms of migration experience excluded by the design. We also considered how our expectation of a woman-vulnerability/man-threat pattern might encourage over-identification of that pattern. Potentially consequential threat matches were consequently reread in context, and the contextual interpretation was retained when it ran against the automated screen.

## 4 Results

### 4.1 Descriptive profile of the corpus

All 900 calls produced usable outputs, divided evenly across the five languages (180 each) and the two gender conditions (450 each). One response departed from the six-sentence instruction; the remaining 99.9% complied. Within-language length differences were small, and none of the five Mann–Whitney comparisons was significant after Holm correction (Table 3). This provides no indication of a substantial length imbalance, although it is not an equivalence test and does not eliminate all possible length-related measurement effects.

**Table 3:** Output length by language and gender.

Language	Measure	Man mean	Woman mean	Difference	Mann–Whitney *p*	Holm *p*
Arabic	words	93.20	93.12	−0.08	0.901	1.000
English	words	103.62	103.38	−0.24	0.954	1.000
Russian	words	91.47	91.08	−0.39	0.553	1.000
Turkish	words	76.03	75.04	−0.99	0.459	1.000
Simplified Chinese	characters	206.60	208.07	+1.47	0.859	1.000

### 4.2 Domain-congruent primary gender comparisons

The common pattern across the primary comparisons was close raw prevalence between the two gender conditions. Agency in achievement scenarios, inclusion in belonging scenarios, and support in care scenarios were all near ceiling. Although prevalence in the woman condition was generally 5.3 percentage points higher across the negative-emotion domains, none of the Fisher’s exact tests was significant, and the Holm-adjusted *p* value was 1.000 for all seven tests. At the same time, the 95% confidence intervals were compatible with differences of roughly 20 percentage points in some comparisons. The results therefore indicate an absence of evidence for a stable gender difference in this sample and configuration, not evidence of equivalence between the gender conditions. Primary gender comparisons are reported in Table 4.

**Table 4:** Prevalence of domain-congruent primary outcomes in the woman and man conditions.

Primary outcome	Man *n*/*N* (%)	Woman *n*/*N* (%)	Difference (pp; 95% CI)	Fisher *p*	Holm *p*
Achievement and agency	71/75 (94.7%)	73/75 (97.3%)	+2.7 [−4.6, +10.5]	0.681	1.000
Belonging and inclusion	73/75 (97.3%)	75/75 (100.0%)	+2.7 [−2.6, +9.2]	0.497	1.000
Loss and sadness	33/75 (44.0%)	37/75 (49.3%)	+5.3 [−10.4, +20.7]	0.624	1.000
Injustice and anger	64/75 (85.3%)	68/75 (90.7%)	+5.3 [−5.4, +16.1]	0.452	1.000
Fear and anxiety	60/75 (80.0%)	64/75 (85.3%)	+5.3 [−6.9, +17.5]	0.518	1.000
Vulnerability	57/75 (76.0%)	61/75 (81.3%)	+5.3 [−7.8, +18.3]	0.550	1.000
Care and support	75/75 (100.0%)	74/75 (98.7%)	−1.3 [−7.2, +3.7]	1.000	1.000

Accounting for the language-scenario strata did not change the primary interpretation. The seven exact stratified *p* values ranged from 0.417 to 1.000, and all were 1.000 after Holm correction. Removing the five-sentence Arabic output and the corresponding opposite-gender condition replicate also left this conclusion unchanged. Complete original and stratified results are provided in Supplementary material S4.

For RQ1 and RQ2, the primary lexical indicators showed no statistically supported gender contrast in the designated domains. This does not establish equivalent emotional roles: lexical presence leaves the experiencer, target, negation, and narrative function unresolved, and the intervals remain compatible with some substantively important differences.

### 4.3 Direction of threat language and counter-framing

The exploratory broad threat-and-suspicion screen identified at least one match in 162/450 man-condition outputs (36.0%) and 121/450 woman-condition outputs (26.9%). The female-minus-male risk difference was −9.1 percentage points (95% CI [−15.1, −3.0]; Fisher *p* = 0.004; Holm *p* = 0.040 across the 10 corpus-wide categories), indicating more frequent matches in the man condition. The exact language-scenario-stratified test also remained significant after the 10-category adjustment (unadjusted *p* = 0.0000815; Holm *p* = 0.000815). These results concern lexical invocation. Threat words can reject a threatening portrayal or refer to danger experienced by the migrant; their frequency cannot establish direct threat attribution.

In TU2, existing explicit threat-negation flags occurred in 16/25 man-condition and 13/25 woman-condition outputs (64% and 52%; unadjusted Fisher *p* = 0.567). Rights or resolution flags occurred in 22/25 and 20/25 outputs (88% and 80%; unadjusted *p* = 0.702). In TU3, explicit threat-negation flags occurred in only 2/25 man-condition and 1/25 woman-condition outputs. Thus, 29 of the 32 negation flags across these two scenarios came from the ticket dispute. These descriptive counts do not support a gender difference in counter-framing, and their concentration cautions against treating it as equally common across situations. Table 5 summarises these existing explicit-negation flags by scenario and gender; it does not classify other forms of threat-related language.

**Table 5:** Existing explicit threat-negation flags by scenario and gender.

Scenario	Man *n*/*N*	Woman *n*/*N*	Total *n*/*N*
TU2: Ticket dispute	16/25	13/25	29/50
TU3: After-dark address	2/25	1/25	3/50
Total	18/50	14/50	32/100

Entries reproduce the existing explicit threat-negation flags in S5. Denominators include all outputs in the specified scenario and gender condition. These flags have not been independently validated and do not provide an exhaustive classification of migrants portrayed as threatening or migrants exposed to danger. An absent flag does not establish either of those categories.

The broad lexical contrast was more widely distributed than the documented negation flags. Male-condition threat matches exceeded female-condition matches in 13 of the 18 scenarios, were equal in two, and were lower in three (Table 6). The largest difference was RI3, dismissive treatment in a public-service setting (19/25 versus 8/25). Omitting any one scenario left a female-minus-male risk difference between −9.9 and −7.1 percentage points. The pooled lexical association was therefore not attributable to a single scenario. Its semantic meaning remains a separate question: the available contextual table does not establish whether this cross-scenario excess reflects threat attribution, threat rejection, danger experienced, or other uses.

**Table 6:** Exploratory broad threat matches by scenario.

Scenario	Man *n*/25	Woman *n*/25	Woman–man (pp)
AA1 Language course	10	8	−8.0
AA2 Promotion	5	3	−8.0
AA3 Helping another migrant	0	1	+4.0
BS1 Neighbourhood meal	2	1	−4.0
BS2 Team success	4	2	−8.0
BS3 Community group	3	0	−12.0
LS1 Family separation	1	0	−4.0
LS2 Ill relative	10	10	+0.0
LS3 Network withdrawal	4	2	−8.0
RI1 Employment discrimination	16	12	−16.0
RI2 Housing discrimination	15	10	−20.0
RI3 Public-service dismissal	19	8	−44.0
TU1 Work-permit uncertainty	12	12	+0.0
TU2 Ticket dispute	22	17	−20.0
TU3 Address search after dark	17	18	+4.0
VC1 Illness while alone	6	5	−4.0
VC2 Lost identity documents	11	6	−20.0
VC3 Workplace accident	5	6	+4.0

Each scenario includes five outputs per language and gender. Entries are lexical matches, not semantic attributions. Counts pool five equally represented languages. The table is descriptive; no scenario-specific confirmatory tests are claimed. Exact scenario wording is in Supplementary material S1. Omitting one scenario at a time leaves female-minus-male risk differences between −9.9 and −7.1 percentage points.

### 4.4 Language-specific exploratory patterns

At the language level, the largest exploratory contrast occurred in Simplified Chinese care scenarios. Vulnerability was lexically detected in 7/15 man outputs (46.7%) and 13/15 woman outputs (86.7%), a difference of 40.0 percentage points. Its two-sided Fisher’s exact *p* value was 0.050. Once the family of 35 exploratory comparisons was Holm-adjusted, however, *p* became 1.000, so the result was not retained. The wording also resisted a simple count. Wúzhù (“helpless”) occurred more often in woman outputs, but several sentences used the term in order to deny it—for example, by stating that she was not seen as helpless. Counting these uses without considering negation overstates the attribution of vulnerability.

Agency terms were more frequent for women in the Russian achievement outputs, as were sadness terms in the English loss outputs. Neither contrast was significant in the unadjusted Fisher tests, and both estimates came from small cells. After correction, none of the 35 language-by-domain comparisons was significant. These exploratory comparisons therefore leave RQ3 unresolved: they provide no evidence of a recurring gender contrast with a consistent direction across languages. Differences in wording observed during focused reading are descriptive and do not establish a language effect. The largest exploratory contrasts are shown in Table 7.

**Table 7:** Largest exploratory contrasts in the language-specific analyses.

Language and domain	Outcome	Man	Woman	Difference (pp)	Fisher *p*	Holm *p*
Chinese—VC	Vulnerability	7/15 (46.7%)	13/15 (86.7%)	+40.0	0.050	1.000
Russian—AA	Agency	11/15 (73.3%)	15/15 (100.0%)	+26.7	0.100	1.000
English—LS	Sadness/loss	10/15 (66.7%)	14/15 (93.3%)	+26.7	0.169	1.000
Chinese—RI	Injustice/anger	11/15 (73.3%)	14/15 (93.3%)	+20.0	0.330	1.000
Chinese—TU	Fear/anxiety	9/15 (60.0%)	12/15 (80.0%)	+20.0	0.427	1.000

### 4.5 Exploratory contextual observations

To address RQ4, both authors jointly examined how counter-framing appeared in the selected outputs. Four interpretive observations emerged from these readings; they do not establish whether the model transforms the broader woman-vulnerability/man-threat binary. First, the event supplied a strong account of the expressed emotion: a verbal disagreement could be described through frustration and rights assertion, while searching for an address after dark could be described through uncertainty. These observations do not establish that scenario effects generally outweigh gender across the corpus.

Second, some outputs preserved migrants’ ordinary needs and capacity to act. Assistance could be temporary, and unfair treatment could coexist with the person’s entitlement to challenge it. This reading identifies possible forms of agency within the selected narratives; no corpus-wide rate of humanising or paternalistic representation was estimated.

Third, some responses explicitly rejected a threatening interpretation. For example, the English TU2 output MAUD-0165 describes a firm response as an attempt to resolve a ticket problem and distinguishes assertiveness from physical danger. We use algorithmic counter-framing as a descriptive label for this textual move: a potentially stereotypical interpretation is made relevant and then constrained or denied. The label denotes an observable discursive construction, not a demonstrated internal model mechanism or a validated new construct.

Fourth, the selected language versions used different formulations to qualify suspicion or defend ordinary behaviour. The released texts allow those formulations to be inspected, but the audit did not estimate the prevalence of distinct rhetorical strategies by language. We therefore make no claim that rights-based, procedural, harmony-oriented, or other formulations characterise an entire language channel or culture.

## 5 Discussion

The seven primary domain-congruent lexical comparisons did not establish a stable gender difference under the tested configuration, including after adjustment for language-scenario strata. The intervals remain too wide to establish equivalence. This differs from the gendered emotion attribution reported by Plaza-del-Arco et al. (2024), but the tasks, models, and measures differ; our result does not overturn their finding. It also cannot establish that the gendered refugee portrayals described by Gray and Franck (2019) and Ryan and Tonkiss (2023) have disappeared from generated migration discourse. The present indicators measure whether selected vocabulary appears, leaving its attribution partly unresolved.

The exploratory threat result illustrates that measurement boundary. The negative female-minus-male risk difference indicates more frequent threat matches in the man condition. This contrast remained after correction across 10 corpus-wide categories and after stratification, and its direction was not driven by one scenario. Yet the same lexical signal can accompany a threatening portrayal, danger experienced by the migrant, or a rejection of the threatening portrayal. The existing contextual flags establish explicit rejection in selected outputs, especially the ticket-dispute scenario. They do not establish that men received more counter-framing across the corpus, or that a lexical difference measures an unequal internal activation of stereotypes.

Algorithmic counter-framing is therefore an exploratory description of particular outputs. In the ticket-dispute example, the text distinguishes firmness from danger; in other selected passages, it separates temporary assistance from permanent helplessness. These constructions may be relevant to representational harm, but their reception and effects were not tested. Narrative studies show why role assignment and social context require attention (Nguyen et al., 2026; Sheikhi, 2026), while the lived-experience validation used in TALES offers a stronger model for future semantic evaluation (Bhagat et al., 2026). A reproducible assessment of counter-framing would need explicit coding rules tested independently across languages and applied to a wider range of scenarios.

For present purposes, emotion terms are not stand-alone measures of a psychological state. The categories associated with Ekman (1992) and the valence-arousal relations proposed by Russell (1980) are helpful for organising the repertoire, but direction changes meaning. Fear experienced by a migrant and fear directed at a migrant occupy very different social relations even when the same term is present. Appraisal theory connects that difference to goals, responsibility, the situation, and perceived coping possibilities (Moors et al., 2013). The EASI model adds the inferences and responses that emotional expression makes available to observers (Van Kleef, 2009). From Barrett's (2006, 2017) constructionist perspective, our concern is not whether the model feels; it is how generated language places culturally available emotion concepts into social roles and moral judgements.

The contribution to emotional-framing research is thus a distinction between lexical invocation and relational attribution. A threat term can position the migrant as the source of another person’s fear, as the person exposed to danger, or as someone being defended against a threatening stereotype. Counts alone cannot distinguish these positions. Equal counts may describe different relations, and differences in counts may arise because the terms serve different purposes. The examples in this audit make that ambiguity visible, while the existing flags quantify only selected instances of explicit negation. They do not validate a measure covering all three relations. An emotion-oriented audit would need to record the person experiencing the emotion, its target, and the role of negation within the narrative as well as the presence of a category term.

Comparisons across languages introduce further uncertainty about measurement. Using a common prompt structure in English, Turkish, Chinese, Russian, and Arabic does not ensure that the resulting indicators measure the same thing. Observed rates may depend on grammar, the availability of equivalent terms, translation choices, or patterns specific to the model’s generation in a language (Kaneko et al., 2022; Ding et al., 2025). In the Chinese care outputs, for example, some vulnerability matches occurred in statements denying helplessness. This interpretive issue remained relevant even though the gender comparison was not significant after correction. Zayed et al. (2024) also report weak agreement between prompt-based fairness measures, underscoring the need to examine what each measure captures. We consequently treat the five channels as linguistic environments in which one model produced text, not as proxies for five cultures.

Both gender conditions contained lexical indicators of agency, care, and belonging. Their representational implications remain limited to the outputs audited here. A service chatbot or automated news system will operate with different users, instructions, institutional goals, and safeguards, and its effects cannot be inferred from these texts. Operational audits should certainly test overt discrimination. They should also examine the less visible work of denial: which stereotype is anticipated, how it is rejected, and whether some language channels repeatedly require that corrective move more than others.

## 6 Limitations

These conclusions are tied to one provider, one model identifier, 1 day of generation, and one set of system instructions. A change in model version, safety alignment, reasoning effort, or prompt wording could alter the distribution of outputs. The present results therefore characterise the configuration tested here. They should not be extended automatically to other generative systems or even to later releases carrying a related model name.

Second, each language-scenario-gender cell contains five independent generations. The total corpus size does not offset this limited within-condition replication; language-specific domain comparisons rely on only 15 outputs per gender. Statistical power for exploratory language differences is therefore limited. Some 95% confidence intervals around the primary risk differences are also compatible with differences of approximately 20 percentage points; nonsignificant *p* values should not be interpreted as evidence of gender equivalence or the absence of substantively important differences. The retrospective precision calculation describes this limitation and does not demonstrate that the original allocation was adequately powered. Future studies should use prespecified equivalence margins, larger numbers of independent generations, and replication across model versions.

Third, the gender manipulation is deliberately minimal and binary. The design does not represent non-binary identities, trans experiences, or intersections between gender and nationality, race, ethnicity, religion, age, class, disability, sexual orientation, or legal status. Holding nationality and legal status outside the prompts makes the gender comparison more controlled, while leaving out intersections that shape migrant representation in actual settings.

Fourth, we elicited responses to brief scenarios written for the study. These texts cannot be treated as samples of news reporting, correspondence from public agencies, or user conversations, and they provide no evidence of audience reception. A model’s correction of a stereotype does not imply that readers would interpret the correction in the same way or that comparable counter-framing would emerge in operational settings.

Fifth, the substring procedure can be reproduced, but its matches have not been validated as measures of meaning. It imposes no word boundaries, uses no language-specific tokenisation or morphological analyser, and retains negated occurrences. Polysemy, inflecxion, segmentation, and unequal lexical coverage may therefore produce different errors across languages. All released indicator fields were reproduced exactly during revision, but this computational check does not establish semantic validity. Neither the lexicons nor the contextual interpretations have independent bilingual validation. The contextual table covers TU2/TU3 and Chinese VC only and does not classify every corpus-wide threat match by direction. These limits prevent estimates of corpus-wide counter-framing prevalence or firm comparisons of rhetorical strategies between languages.

Finally, repeated model calls are not independent human observations, nor does sharing a replicate number make woman and man outputs statistically paired generations. The stratified tests characterise stochastic variation within fixed language-scenario conditions; they do not provide probabilistic generalisation to human populations, other models, or future versions. The explicitly directional structure of some scenarios may also have produced ceiling effects in agency, belonging, and care. These distinctions are particularly important in preventing small *p* values from being interpreted as evidence of universal model behaviour.

## 7 Conclusion

This audit of 900 outputs across five languages found no statistically supported gender difference in the seven primary domain-congruent lexical outcomes under the tested model and prompt configuration. The conclusion was unchanged by exact language-scenario-stratified comparisons. The intervals remain too wide to establish equivalence, and five generations per condition limit sensitivity, particularly within languages.

The exploratory broad threat screen showed a male-condition excess that remained after correction across 10 categories and was distributed across several scenarios. This is a lexical association, not evidence that male migrants were more often represented as dangerous. Existing contextual flags documented explicit threat rejection mainly in the ticket-dispute scenario. These outputs motivate the exploratory concept of algorithmic counter-framing, but do not establish its corpus-wide prevalence, a gender asymmetry in its use, or its effects on readers.

The ticket-dispute passages illustrate the value of examining how a response qualifies its own description. Firmness may be acknowledged while an inference of danger is rejected. Such wording leaves the stereotype available within the account even as it disputes its application to the person described. Whether readers experience that move as corrective, patronising, or as a reminder of the stereotype remains an empirical question. The present output audit cannot resolve it.

For research on emotional framing, the findings show why recording a word and identifying the relation it expresses are separate analytical tasks. Fear-related language can designate a migrant as a source of danger, a person experiencing danger, or someone defended against a stereotype. Recording who experiences the emotion, toward whom it is directed, and how the narrative qualifies it would make multilingual audits more informative. Independent semantic validation is needed before these distinctions can support broader claims about gendered representation or social inclusion.

The multilingual design also makes the limits of comparison visible. Holding scenarios and gender cues constant provided a common basis for generating texts, but did not settle whether lexical matches carried comparable meanings in each language. The Chinese instances that denied helplessness are particularly instructive here: their inclusion in a vulnerability count changes what that count can reasonably support. Language-specific interpretation therefore remains necessary even when the experimental structure is balanced.

Further work could build on the released materials by applying explicit relational coding to all threat matches, using independent multilingual assessment and more repetitions within conditions. Replication across models and prompt settings would then show how far the observed patterns extend. Studies of readers would address a different question: whether these portrayals affect recognition or judgements of migrants. Together, these steps would connect reproducible output comparisons with the social consequences that motivate migration communication research.

## Data Availability

The original contributions presented in the study are included in the article/Supplementary material, further inquiries can be directed to the corresponding authors.
