# Supplementary material for: Who is feared and who is protected? A multilingual audit of gendered emotional framing of migrants in generative AI
Source: Front Psychol. 2026 Oct 27;17:1980883. doi: 10.3389/fpsyg.2026.1980883 (PMC13748444; doi:10.3389/fpsyg.2026.1980883)
Supplement: Supplementary file 1 [file Data_Sheet_1.ZIP › Supplementary Files/Supplementary_Material_S1-S8_Revised.docx]

Supplementary Material

Who Is Feared and Who Is Protected? A Multilingual Audit of Gendered Emotional Framing of Migrants in Generative AI

Ayhan Dolunay & Fatma Arpalıklı
Near East University, Nicosia, Cyprus

This document accompanies the machine-readable supplementary package. It reports the original design and descriptive analyses (S1–S7) together with the additional sensitivity and transparency analyses prepared during revision (S8). The package contains the exact prompts, all 900 original model outputs, five clearly identified language-specific subsets derived from the merged corpus, the original released lexical indicators and lexicons, executable analysis code, statistical result files and the stored focused contextual-audit records. No external repository deposit or independent semantic validation is implied.

# S1. Experimental design and corpus generation

The audit used a balanced controlled design in which gender description was the manipulated factor. Each scenario was generated independently in five languages, with five stochastic outputs for each language-scenario-gender condition. No response-level pairing or common sampling seed linked the woman and man calls.

| **Factor** | **Levels** | **Count / contribution** |
| --- | --- | --- |
| Language | English, Turkish, Simplified Chinese, Russian, Modern Standard Arabic | 5; 180 outputs per language |
| Thematic domain | AA, BS, LS, RI, TU, VC | 6; 150 outputs per domain |
| Scenario | Three scenarios per domain | 18; 50 outputs per scenario |
| Gender condition | Woman migrant / man migrant | 2; 450 outputs per condition |
| Replicate | Independent stochastic generations | 5 per language-scenario-gender cell |
| Total |  | 900 outputs |

Generation was performed on 27 August 2026 through the OpenAI Responses API using gpt-5.6-terra, medium reasoning effort, medium text verbosity, and a 2,048-token maximum. Temperature and top_p were not separately set. The exact system instructions, scenario texts, task instructions, and assembled user prompts are supplied in S1_Prompt_Manifest_180_Conditions.csv.

## S1.1. Scenario inventory

| **ID** | **Domain** | **Domain label** | **English gender-neutral scenario core** |
| --- | --- | --- | --- |
| AA1 | AA | Achievement and Agency | Completes an advanced local-language course and gives a short ceremony speech. |
| AA2 | AA | Achievement and Agency | Receives a promotion after two years of strong work performance. |
| AA3 | AA | Achievement and Agency | Helps a newly arrived migrant with forms, transport, and basic procedures. |
| BS1 | BS | Belonging and Safety | Is invited to a shared neighborhood meal by several neighbors. |
| BS2 | BS | Belonging and Safety | Has a contribution publicly recognized after a successful team project. |
| BS3 | BS | Belonging and Safety | Joins a local community group for the first time. |
| LS1 | LS | Loss and Separation | Joins an important family celebration remotely by video call. |
| LS2 | LS | Loss and Separation | Cannot reach a seriously ill relative because of work and administrative constraints. |
| LS3 | LS | Loss and Separation | Moves to another city for a stable job, leaving a close social network behind. |
| RI1 | RI | Rejection and Injustice | Is rejected for a job after attention shifts to accent and origin. |
| RI2 | RI | Rejection and Injustice | Is rejected for housing after the landlord learns the applicant is a migrant. |
| RI3 | RI | Rejection and Injustice | Receives dismissive and unequal treatment in a public-service setting. |
| TU1 | TU | Threat and Uncertainty | Waits for a decision on an application that determines whether work can continue. |
| TU2 | TU | Threat and Uncertainty | Speaks firmly with staff about a ticket problem in a crowded station; the dispute remains verbal. |
| TU3 | TU | Threat and Uncertainty | Searches for an address after dark while a resident watches from a short distance. |
| VC1 | VC | Vulnerability and Care | Becomes ill while living alone; a neighbor offers food and help arranging medical care. |
| VC2 | VC | Vulnerability and Care | Loses a wallet containing important identity documents while using public transport. |
| VC3 | VC | Vulnerability and Care | Has a temporary workplace injury; coworkers organize practical assistance during recovery. |

# S2. Corpus integrity and provenance

The merged original corpus contains 900 records and 37 source variables, with 180 outputs per language. Five language-specific CSV files now included in the package were deterministically extracted from this merged corpus for convenient review. Each extracted subset was checked against the merged file by output identifier and exact output text. They are derived subsets, not separately archived independent original sources. The complete prompt manifest was checked against the 900 raw records for exact system instruction, scenario text, task instruction and assembled user prompt.

| **Check** | **Observed** | **Expected** | **Status** |
| --- | --- | --- | --- |
| Total records | 900 | 900 | PASS |
| Unique output_id | 900 | 900 | PASS |
| Unique run_key | 900 | 900 | PASS |
| Empty output_text | 0 | 0 | PASS |
| Non-success status | 0 | 0 | PASS |
| Non-completed response_status | 0 | 0 | PASS |
| Retry count > 0 | 0 | 0 | PASS |
| Six-sentence outputs | 899 | 899 | PASS |
| Five-sentence outputs | 1 | 1 | PASS |

The single five-sentence output is MAUD-0849 (AR-LS3-M-R3); it was retained in the main analysis. The raw corpus is otherwise complete, with no empty outputs, failed calls, duplicate condition keys, or retries. Total recorded usage was 236,350 input tokens and 151,723 output tokens. Collection ran from 02:16:06 to 03:16:36 UTC.

# S3. Multilingual coding framework

Ten overlapping categories were screened using 554 language–category literal patterns (English 117, Turkish 109, Simplified Chinese 119, Russian 99, Arabic 110). After Unicode NFKC normalization and lowercasing, patterns were matched as literal substrings. Arabic processing additionally removed diacritics and elongation marks and standardized listed letter variants. The procedure used neither word-boundary restrictions nor automatic linguistic tokenization, stemming, lemmatization or Chinese segmentation. Some patterns were manually listed as stems. Category presence and the number of distinct matched patterns were retained for each response. Negated matches remained positive lexical indicators.

| **Category** | **Analytical focus** | **Typical indicators** |
| --- | --- | --- |
| Fear and anxiety | Uncertainty and high-arousal negative affect | fear, anxiety, worry, tension, unease |
| Anger and injustice | Emotional and moral response to unfair treatment | anger, discrimination, injustice, humiliation, exclusion |
| Sadness and loss | Separation, loneliness, and loss of connection | sadness, longing, loneliness, grief, loss |
| Positive affect | Positive valence and future orientation | pride, joy, hope, relief, gratitude |
| Vulnerability | Powerlessness, dependency, or susceptibility to harm | vulnerability, helplessness, isolation, dependency |
| Care and support | Relational or institutional assistance | help, care, empathy, protection, solidarity |
| Agency and competence | Autonomy, skill, and problem-solving capacity | determination, competence, achievement, initiative, resilience |
| Belonging and inclusion | Acceptance, participation, and social connection | belonging, welcome, membership, recognition, safety |
| Threat and suspicion | Danger, doubt, or security language | threat, danger, suspicion, caution, aggression |
| Family and attachment | Family members and emotional closeness | family, relative, close person, loved one |

The complete lexicons are in S3_Multilingual_Lexicons.csv, with precise rules in S3_Coding_Rules.txt and all original coded indicators in S3_Coded_Indicators_900.csv. Prompts and lexical lists were first developed in Turkish and English; Simplified Chinese, Russian and Modern Standard Arabic versions were translated and subsequently checked. Generative AI assisted prompt development and translation. These preparation checks are not documented independent bilingual validation. The data record lexical invocation; a match does not establish whose emotion is expressed, its target, its direction, or semantic equivalence across languages.

## S3.1. Domain-congruent primary coding

| **Domain** | **Primary category** |
| --- | --- |
| AA – Achievement and Agency | Agency and competence |
| BS – Belonging and Safety | Belonging and inclusion |
| LS – Loss and Separation | Sadness and loss |
| RI – Rejection and Injustice | Anger and injustice |
| TU – Threat and Uncertainty | Fear and anxiety |
| VC – Vulnerability and Care | Vulnerability |
| VC – Vulnerability and Care | Care and support |

# S4. Statistical analysis

The original analysis treated each API call as one stochastic output and used two-sided Fisher exact tests, female-minus-male risk differences with 95% Newcombe-Wilson confidence intervals, Holm correction for seven domain-congruent primary comparisons, Mann-Whitney U tests for within-language output lengths (five-test Holm family), and a separate exploratory family of 35 language-by-domain comparisons. During revision, exact conditional tests stratified jointly by language and scenario were added (15 strata for each primary comparison, 90 for each corpus-wide category). Within each stratum, the female positive count was modeled by the hypergeometric distribution conditional on that stratum’s total positives. Convolution of the within-stratum null distributions yielded an exact two-sided p value based on absolute distance of the summed count from its conditional expectation. The seven stratified primary tests were Holm-adjusted separately. The corpus-wide ten-category screen is explicitly exploratory: ten pooled tests and ten stratified tests were each corrected within their own ten-test families. Neither these revision analyses nor the precision benchmark were preregistered or used to determine the original five-replicate design. The original unstratified marginal estimates and confidence intervals are retained for description; nonsignificance is not an equivalence finding.

## S4.1. Primary domain-congruent comparisons

| **Primary outcome** | **Man n/N (%)** | **Woman n/N (%)** | **Difference (pp; 95% CI)** | **Fisher p** | **Holm p** |
| --- | --- | --- | --- | --- | --- |
| Achievement and agency | 71/75 (94.7%) | 73/75 (97.3%) | +2.7 [-4.6, +10.5] | 0.681 | 1.000 |
| Belonging and inclusion | 73/75 (97.3%) | 75/75 (100.0%) | +2.7 [-2.6, +9.2] | 0.497 | 1.000 |
| Loss and sadness | 33/75 (44.0%) | 37/75 (49.3%) | +5.3 [-10.4, +20.7] | 0.624 | 1.000 |
| Injustice and anger | 64/75 (85.3%) | 68/75 (90.7%) | +5.3 [-5.4, +16.1] | 0.452 | 1.000 |
| Fear and anxiety | 60/75 (80.0%) | 64/75 (85.3%) | +5.3 [-6.9, +17.5] | 0.518 | 1.000 |
| Vulnerability | 57/75 (76.0%) | 61/75 (81.3%) | +5.3 [-7.8, +18.3] | 0.550 | 1.000 |
| Care and support | 75/75 (100.0%) | 74/75 (98.7%) | -1.3 [-7.2, +3.7] | 1.000 | 1.000 |

## S4.2. Output length comparisons

| **Language** | **Measure** | **Man mean** | **Woman mean** | **Difference** | **Mann-Whitney p** | **Holm p** |
| --- | --- | --- | --- | --- | --- | --- |
| Modern Standard Arabic | words | 93.20 | 93.12 | -0.08 | 0.901 | 1.000 |
| English | words | 103.62 | 103.38 | -0.24 | 0.954 | 1.000 |
| Russian | words | 91.47 | 91.08 | -0.39 | 0.552 | 1.000 |
| Turkish | words | 76.03 | 75.04 | -0.99 | 0.459 | 1.000 |
| Simplified Chinese | characters | 206.60 | 208.07 | +1.47 | 0.859 | 1.000 |

## S4.3. Largest language-specific exploratory contrasts

| **Language-domain** | **Outcome** | **Man** | **Woman** | **Difference (pp)** | **Fisher p** | **Holm p** |
| --- | --- | --- | --- | --- | --- | --- |
| ZH – VC | Vulnerability | 7/15 | 13/15 | +40.0 | 0.050 | 1.000 |
| RU – AA | Achievement and agency | 11/15 | 15/15 | +26.7 | 0.100 | 1.000 |
| EN – LS | Loss and sadness | 10/15 | 14/15 | +26.7 | 0.169 | 1.000 |
| ZH – RI | Injustice and anger | 11/15 | 14/15 | +20.0 | 0.330 | 1.000 |
| ZH – TU | Fear and anxiety | 9/15 | 12/15 | +20.0 | 0.427 | 1.000 |

All 35 exploratory comparisons are in S4 Analysis/Original_Results_Reproduced/reproduced_exploratory_35_results.csv. The added exact stratified and corpus-wide revision calculations and their complete multiple-comparison families are in S4 Analysis/Revision_Results; summary tables appear in Section S8 below.

# S5. Threat direction and contextual validation

The exploratory broad threat-and-suspicion screen matched 162/450 man-condition outputs (36.0%) and 121/450 woman-condition outputs (26.9%): a man-condition excess of 9.1 percentage points. The female-minus-male Newcombe-Wilson 95% confidence interval is [−15.1, −3.0] percentage points; the pooled Fisher p value is 0.004041 and the revision Holm-adjusted pooled p value across ten categories is 0.040413. The ten-category-adjusted language–scenario-stratified exact p value is 0.000815. These are lexical-match frequencies and cannot be interpreted as direct threat attribution.

The released S5 contextual table contains all 100 ticket-dispute (TU2) and after-dark address-search (TU3) outputs across the five languages, and all 30 Simplified Chinese vulnerability-and-care outputs. It preserves original output text and stored automated flags for explicit threat negation, rights/resolution language and identity/behavior decoupling. Existing explicit threat-negation flags total 29/50 in TU2 (16/25 man, 13/25 woman) and 3/50 in TU3 (2/25 man, 1/25 woman). A missing flag does not establish positive threat attribution. The original code that generated these stored contextual flags was not supplied; the present package verifies their counts but cannot independently regenerate or validate the flags. No comprehensive three-way coding of the 283 threat-match outputs, independent multilingual contextual validation, or gender difference in counter-framing is claimed.

| **Focused check** | **Man** | **Woman** | **Interpretation** |
| --- | --- | --- | --- |
| TU2 explicit threat negation | 16/25 | 13/25 | Existing stored explicit-negation flag (not independently re-generated) |
| TU2 rights/resolution language | 22/25 | 20/25 | Existing rights/resolution screen; not independent semantic validation |
| ZH-VC lexical vulnerability | 7/15 | 13/15 | Exploratory lexical result; Holm-adjusted p = 1.000 within 35 comparisons |
| ZH-VC 无助 (“helpless”) | 5/15 | 10/15 | Literal lexical presence, including negated uses; not a positive attribution rate |

# S6. Reproducibility file map

| Package root | README_Revision.txt (execution, literal file map and provenance); verify_package.py (offline end-to-end checks); requirements.txt (dependencies); Supplementary_Material_S1-S8_Revised.docx (this document) |
| --- | --- |
| S1 Design and Prompts | S1_Generation_Code.py, S1_Scenario_Manifest_18.csv and S1_Prompt_Manifest_180_Conditions.csv (exact released prompts) |
| S2 Audit Corpus | Original S2_Complete_Audit_Corpus_900.csv and five derived per-language 180-record CSV subsets |
| S3 Coding | Original multilingual lexicons, rules and released response-level indicators |
| S4 Analysis | Original S4_Analysis_Code.py (paths corrected), original XLSX summary, Original_Results_Reproduced CSVs, S4_Revision_Analysis.py and Revision_Results CSVs |
| S5 Contextual Audit | Original S5_Contextual_Audit.csv and clarified codebook. Original generation code for these stored contextual flags is not available. |
| S8 (within this document) | Additional revision Tables S8.1–S8.5. The underlying machine-readable analyses are in S4 Analysis/Revision_Results. |

# S7. Data availability statement consistent with the submitted package

The complete 900-output synthetic audit corpus, exact prompts and condition identifiers, generation metadata, five language-specific subsets derived from the merged corpus, language-specific lexicons, matching rules, response-level coded indicators, code reproducing the original lexical and statistical analyses, added revision analyses, scenario-level summaries and stored focused contextual-audit records are provided as Supplementary Material for review. An execution README and output check list are included. These files do not constitute a public repository deposit or independent bilingual semantic validation. The original code for deriving the stored contextual flags is not available, so only their recorded counts, not their generation process, can be reproduced.

S8. Additional revision analyses and transparency tables

All results in this section were calculated during revision. They are not represented as prespecified or preregistered analyses. The exact machine-readable results, executable code and assumptions are included in S4 Analysis/Revision_Results and S4_Revision_Analysis.py. Risk differences below are WOMAN MINUS MAN; a negative value indicates higher lexical occurrence in the man condition.

## S8.1. Primary outcome contrasts: pooled and stratified tests

Table S8.1 reports the seven domain-congruent comparisons (75 outputs per gender per domain). The original pooled Fisher and the added stratified exact tests have separate seven-test Holm corrections. All stratified adjusted p values equal 1.000; the pooled risk differences remain marginal descriptions of the recorded corpus.

| Outcome | Man n/75 | Woman n/75 | Woman − man (pp) | Pooled Fisher p | Pooled Holm-7 | Strat. exact p | Strat. Holm-7 |
| --- | --- | --- | --- | --- | --- | --- | --- |
| Achievement and agency | 71 | 73 | 2.7 | 0.681 | 1.000 | 0.673 | 1.000 |
| Belonging and inclusion | 73 | 75 | 2.7 | 0.497 | 1.000 | 0.444 | 1.000 |
| Loss and sadness | 33 | 37 | 5.3 | 0.624 | 1.000 | 0.512 | 1.000 |
| Injustice and anger | 64 | 68 | 5.3 | 0.452 | 1.000 | 0.417 | 1.000 |
| Fear and anxiety | 60 | 64 | 5.3 | 0.518 | 1.000 | 0.481 | 1.000 |
| Vulnerability | 57 | 61 | 5.3 | 0.550 | 1.000 | 0.536 | 1.000 |
| Care and support | 75 | 74 | -1.3 | 1.000 | 1.000 | 1.000 | 1.000 |

The original pooled female-minus-male Newcombe-Wilson 95% intervals and full-precision results remain in the original reproduced CSVs; R2_Primary_Stratified.csv records these intervals and conditional null expectations.

## S8.2. All ten corpus-wide lexical comparisons (exploratory)

Table S8.2 documents the full exploratory multiplicity family. Both pooled Fisher and language–scenario-stratified exact tests were Holm-adjusted across all ten categories, separately by method; this does not make them confirmatory tests. Direction is woman minus man. A threat match does not identify a threatening portrayal.

| Category | Man /450 | Woman /450 | Woman − man pp | Fisher p | Fisher Holm-10 | Strat. p | Strat. Holm-10 |
| --- | --- | --- | --- | --- | --- | --- | --- |
| agency competence | 299 | 303 | 0.9 | 0.832 | 1.000 | 0.755 | 1.000 |
| anger injustice | 83 | 88 | 1.1 | 0.734 | 1.000 | 0.508 | 1.000 |
| belonging inclusion | 279 | 280 | 0.2 | 1.000 | 1.000 | 1.000 | 1.000 |
| care support | 328 | 348 | 4.4 | 0.143 | 1.000 | 0.037 | 0.332 |
| family attachment | 170 | 167 | -0.7 | 0.890 | 1.000 | 0.792 | 1.000 |
| fear anxiety | 248 | 240 | -1.8 | 0.640 | 1.000 | 0.531 | 1.000 |
| positive affect | 227 | 220 | -1.6 | 0.689 | 1.000 | 0.566 | 1.000 |
| sadness loss | 82 | 78 | -0.9 | 0.794 | 1.000 | 0.693 | 1.000 |
| threat suspicion | 162 | 121 | -9.1 | 0.004 | 0.040 | 0.000081 | 0.000815 |
| vulnerability | 136 | 148 | 2.7 | 0.430 | 1.000 | 0.303 | 1.000 |

## S8.3. Scenario-level threat vocabulary and leave-one-scenario-out checks

Table S8.3 lists each of the 18 scenarios (25 outputs per gender). The lexical match rate for the man condition exceeded that for the woman condition in 13 scenarios, was equal in two and was lower in three. Excluding any individual scenario yields a man-condition excess of 7.1–9.9 percentage points. These facts describe the breadth of the lexical result ONLY; the separately stored explicit-negation flags are concentrated in TU2.

| Scenario | Man /25 | Woman /25 | Woman − man (pp) |
| --- | --- | --- | --- |
| AA1 | 10 | 8 | -8.0 |
| AA2 | 5 | 3 | -8.0 |
| AA3 | 0 | 1 | 4.0 |
| BS1 | 2 | 1 | -4.0 |
| BS2 | 4 | 2 | -8.0 |
| BS3 | 3 | 0 | -12.0 |
| LS1 | 1 | 0 | -4.0 |
| LS2 | 10 | 10 | 0.0 |
| LS3 | 4 | 2 | -8.0 |
| RI1 | 16 | 12 | -16.0 |
| RI2 | 15 | 10 | -20.0 |
| RI3 | 19 | 8 | -44.0 |
| TU1 | 12 | 12 | 0.0 |
| TU2 | 22 | 17 | -20.0 |
| TU3 | 17 | 18 | 4.0 |
| VC1 | 6 | 5 | -4.0 |
| VC2 | 11 | 6 | -20.0 |
| VC3 | 5 | 6 | 4.0 |

Full leave-one-scenario-out values, including the remaining counts and denominator 425 per gender, are supplied in R4_Leave_One_Scenario_Out.csv; detailed scenario totals are supplied in R3_Threat_by_Scenario.csv.

## S8.4. Coverage and recorded flags in the focused contextual audit

Table S8.4 reports only information preserved in the existing S5 file or in the original deterministic S3 indicators. The 100 TU2/TU3 records cover both gender conditions across all five languages; a separate set of 30 Simplified Chinese VC records was also released. TU2 and TU3 flag totals are NOT the three requested comprehensive narrative categories. An unflagged record must not be read as portraying a migrant as dangerous, and the original source procedure for producing stored S5 flags is not in the archive.

| Focused measure | Man flagged /N | Woman flagged /N | Data source |
| --- | --- | --- | --- |
| TU2 explicit threat-negation flag | 16/25 | 13/25 | stored S5 flags |
| TU2 rights or resolution flag | 22/25 | 20/25 | stored S5 flags |
| TU3 explicit threat-negation flag | 2/25 | 1/25 | stored S5 flags |
| ZH-VC lexical vulnerability presence | 7/15 | 13/15 | S3 coded indicators |
| ZH-VC lexical 无助 | 5/15 | 10/15 | S3 coded indicators |

Recorded explicit threat-negation totals: 29/50 in TU2 (16/25 man; 13/25 woman) and 3/50 in TU3 (2/25 man; 1/25 woman). Twenty-nine of the 32 recorded flags across these two scenarios are concentrated in TU2. They do not establish corpus-wide prevalence or a gender difference in algorithmic counter-framing. In TU2, unadjusted two-sided Fisher tests on the stored flags yield p = 0.567 for explicit negation and p = 0.702 for rights/resolution; they do not establish a gender difference.

## S8.5. Retrospective precision benchmark for five replicates per cell

Five independent generations per language–scenario–gender cell were selected as an author design judgment, not by an a priori power or precision calculation. Under an idealized independent-binomial approximation with probability 0.50 in each gender, the approximate 95% difference-interval half-width is 1.96 × sqrt(0.5/n), where n is the number of responses per gender. This retrospective illustration must not be interpreted as observed power or evidence that the design had adequate sensitivity.

| Analysis unit | n per gender | Assumed p per gender | Approx. 95% half-width (pp) |
| --- | --- | --- | --- |
| primary pooled domain; per gender | 75 | 0.5 | 16.0 |
| exploratory language-domain; per gender | 15 | 0.5 | 35.8 |

Data and code provenance: the original corpus, prompts, lexicons, released lexical indicators and stored contextual records were preserved unchanged. Only derived per-language subsets, the corrected original analysis script, generated original-statistics reproductions, newly added revision-statistics code and results, the clarified S5 codebook, and this supplementary document were added or modified. S5 original contextual-flag generation remains unavailable.
